# The ‘autoimmunome’ of centenarians

**DOI:** 10.1016/j.jtauto.2025.100295

**Published:** 2025-06-07

**Authors:** Pedro Carrera-Bastos, Abel Plaza-Florido, Alejandro Santos-Lozano, Vânia Borba, Gabriel Rodríguez-Romo, Celia García-Chico, Simone Lista, Gonzalo Saco-Ledo, Enzo Emanuele, Yehuda Shoenfeld, Alejandro Lucia

**Affiliations:** a Faculty of Biomedical and Health Sciences. Universidad Europea de Madrid, Madrid, Spain; b Center for Primary Health Care Research, Department of Clinical Sciences, Lund University, Malmö, Sweden; c Rio Maior School of Sport - Santarém Polytechnic University, Rio Maior, Portugal; d Research Center for Exercise Medicine and Sleep/Pediatric Exercise and Genomics Research Center, Department of Pediatrics, School of Medicine, University of California Irvine, Irvine, CA, USA; e i+HeALTH Strategic Research Group, Department of Health Sciences, Miguel de Cervantes European University, Valladolid, Spain; f Research Institute of the Hospital 12 de Octubre (‘imas12’), Madrid, Spain; g Internal Medicine, Rehaklinik Dussnang, Thurgau, Switzerland; h Deporte y Entrenamiento Research Group, Departamento de Deportes, Faculty of Physical Activity and Sport Sciences (INEF), Universidad Politécnica de Madrid, Madrid, Spain; i Centro de Investigación Biomédica en Red de Fragilidad y Envejecimiento Saludable (CIBERFES), Instituto de Salud Carlos III, Madrid, Spain; j Department of Sports Sciences. Faculty of Medicine, Health and Sports. Universidad Europea de Madrid, Madrid, Spain; k 2E Science, Pavia, Italy; l Zabludowicz Center for Autoimmune Diseases, Sheba Medical Center, Tel-Hashomer, Israel; m Dina Recanati School of Medicine, Reichman University, Herzliya, Israel

**Keywords:** Autoimmunity, Aging, Longevity, Protein-protein interaction, Complement system, Centenarians

## Abstract

**Objective:**

To identify signature proteins potentially linked to resistance to autoimmunity in the blood of centenarians.

**Methods:**

We conducted *in silico* data mining of previously published proteomic results using the Search Tool for the Retrieval of Interacting Genes/Proteins (STRING) and PHENOPEDIA databases.

**Results:**

Sixteen autoimmune disease-related proteins were identified within the proteomic signatures of centenarians. Albumin was the most connected hub protein, notably elevated in centenarians compared to younger controls, suggesting a protective role. Eight of the identified autoimmunity-related proteins—ADIPOQ, C1S, C5, C7, C9, CFD, MASP1, and SERPING1—were associated with the complement system.

**Conclusion:**

Elevated albumin levels and a prominent complement system presence in centenarians' blood proteome may contribute to resistance to autoimmunity, highlighting potential protective mechanisms against autoimmune diseases in extreme longevity.

## Graphical abstract

Graphical abstract summarizing the identification of autoimmune disease (AD)-related proteins in the blood proteome of centenarians. Proteomic data from two independent studies were analyzed using liquid chromatography-tandem mass spectrometry (LC-MS/MS) and SomaScan platforms. *In silico* data mining using Search Tool for the Retrieval of Interacting Genes/Proteins (STRING) and PHENOPEDIA identified 16 AD-related proteins. ALB (albumin) emerged as the central hub in the protein–protein interaction (PPI) network and was notably elevated in centenarians compared to younger controls. Eight proteins (see text for symbol explanations)—ADIPOQ, C1S, C5, C7, C9, CFD, MASP1, and SERPING1—were associated with the complement system, supporting its potential role in resistance to autoimmunity in extreme longevity. Created with BioRender.com. **Note:** Protein identifiers are based on HGNC (HUGO Gene Nomenclature Committee) gene symbols, as used in proteomics and bioinformatics resources such as STRING or PHENOPEDIA. While these symbols technically denote genes, they are used here to represent proteins and are formatted in non-italicized uppercase, in line with standard practice in the field.

**Figure:**
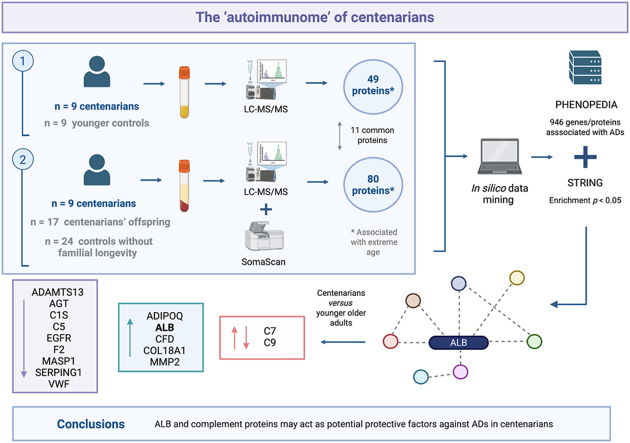

## 1 Introduction

Autoimmune diseases (ADs) result from a breakdown of immune tolerance [1], which in turn can be exacerbated by two age-related conditions: immunosenescence and low-grade chronic systemic inflammation (‘inflammaging’) [[2], [3], [4]]. Accordingly, age is associated with an increased susceptibility to (and severity of) ADs such as rheumatoid arthritis [5], autoimmune thyroid disorders [6], or multiple sclerosis [7]. Yet, despite frequently having circulating autoantibodies, centenarians (*i.e.*, individuals aged 100+ years) rarely develop clinical ADs [8]. This apparently paradoxical observation points toward adaptive immune mechanisms in these individuals, including IgM autoantibodies with protective functions, enhanced regulatory T-cell responses, or distinct genetic and epigenetic signatures [8,9].

Proteomic analyses provide comprehensive protein interaction maps that highlight key pathways and molecular interactions, thereby contributing to our understanding of disease processes [10]. Previous work by our group [11] and others [12] identified specific proteomic signatures in the blood of centenarians compared to (younger) older adults, prompting further investigation specifically targeting proteins related to ADs in extreme longevity.

The term “autoimmunome” was originally used to describe the full set of molecular, genetic, and regulatory components implicated in AD susceptibility and pathogenesis [13]. Here, we extend this concept to refer specifically to the proteomic network associated with resistance to autoimmunity in centenarians.

## 2 Materials and methods

This study involved *in silico* data mining of previously published proteomic results [11,12] using the Search Tool for the Retrieval of Interacting Genes/Proteins (STRING) [14] and the PHENOPEDIA database [15]. We used as input an 80-protein signature associated with extreme age identified in the serum of 9 centenarians (5 women, mean age 101.9 years) compared to 17 centenarians' offspring (9 women, mean age 70.6 years) and 24 controls without familial longevity (14 women, mean age 71.5 years), through two different proteomic platforms: liquid chromatography-tandem mass spectrometry (LC-MS/MS) and the SomaScan array. [12]. Additionally, we included 49 proteins that were differentially expressed (as shown by LC-MS/MS) in the plasma of 9 disease-free centenarians (5 women, 100–103 years) compared to 9 younger controls (5 women) who had died (at age 67–81 years) from major age-related diseases [11]. Eleven proteins overlapped in both datasets: afamin (AFM), beta-2-microglobulin (B2M), cartilage acidic protein 1 (CRTAC1), complement component 7 (C7) and 9 (C9), cystatin C (CST3), EGF-containing fibulin-like extracellular matrix protein 1 (EFEMP1), insulin-like growth factor-binding protein complex acid labile subunit (IGFALS), inter-alpha-trypsin inhibitor heavy chain H3 (ITIH3), lysozyme (LYZ), and serpin family F member 2 (SERPINF2) [11,12].

Protein-protein interaction (PPI) networks were explored using the STRING database, applying stringent parameters (interaction score ≥ 0.90, PPI enrichment *p* < 0.05) to ensure high-confidence interactions. AD-related proteins were assessed using STRING's functional annotations and the PHENOPEDIA database [15]. The latter includes 946 genes/proteins associated with ADs. This dual-annotation strategy provided robust support for the relevance of the proteins identified in the centenarians' blood proteome in the context of ADs.

Throughout this article, we used HGNC (HUGO Gene Nomenclature Committee) gene symbols to denote proteins. Although these symbols are technically gene identifiers, they are widely adopted in proteomics and bioinformatics platforms (e.g., STRING, PHENOPEDIA) as standard shorthand for the relevant protein products. Accordingly, we used HGNC symbols in non-italicized uppercase format to represent proteins—rather than genes—unless otherwise specified.

## 3 Results

**Fig. 1:**
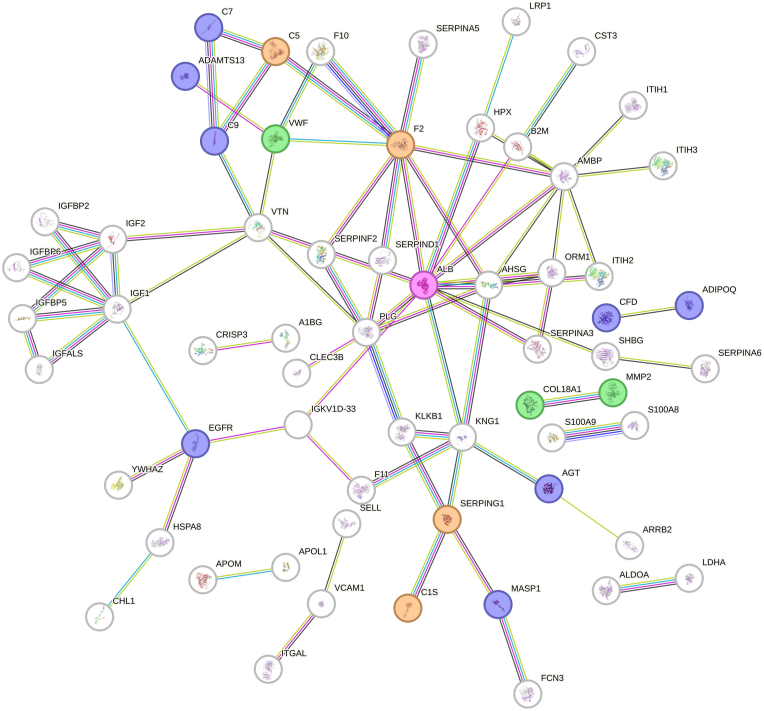
Protein-protein interaction (PPI) network of centenarians, comprising 118 nodes (disconnected nodes are hidden) and 81 edges (expected = 6, PPI enrichment *p* < 1.0 x 10^−16^), with an average node degree of 1.38 and an average local clustering coefficient of 0.335. Colored nodes indicate proteins potentially associated with autoimmune diseases: green (identified only via Search Tool for the Retrieval of Interacting Genes/Proteins (STRING) functional annotation), violet (identified only in the PHENOPEDIA database), orange (identified in both databases), and pink (protein with the highest node degree).

Our analysis identified 16 proteins within the centenarians’ blood proteome network that are associated (whether directly or inversely) with ADs (Fig. 1 and Table 1).

**Table 1:** Proteins identified in the protein-protein interaction (PPI) network of centenarians and their role in autoimmune diseases (ADs).

Gene symbol^a^	Regulation in centenarians versus younger controls	Association with ADs
ADAMTS13	↓ [12]	This protein attenuates inflammatory responses in experimental autoimmune encephalomyelitis [16] and has been associated with acquired thrombotic thrombocytopenic purpura [17].
ADIPOQ	↑ [12]	ADIPOQ is dysregulated in systemic autoimmune rheumatic diseases [18].
ALB	↑ [11]	Higher ALB levels are inversely associated with the severity of certain ADs [[19], [20], [21]].
AGT	↓ [12]	This precursor of angiotensin II is involved in the progression (and associated inflammation) of rheumatoid arthritis, systemic lupus erythematosus, and multiple sclerosis [22]. Angiotensin II blockers can reduce inflammation in rheumatic diseases [22].
C5	↓ [11]	The suppression of C5 production has been proposed as a potent therapeutic strategy for ADs [23].
C7^b^	↓ [11] ↑ [12]	C7 deficiency is associated with increased susceptibility to Neisseria infections and with systemic lupus erythematosus [24].
C9^b^	↓ [11] ↑ [12]	C9 deficiency has been associated with Behçet's disease [25].
C1S	↓ [11]	Patients with systemic lupus erythematosus show higher plasma levels of C1s compared with controls [26].
CFD	↑ [12]	Deficiency of complement factor D has been associated with an increased susceptibility to ADs [27].
COL18A1	↑ [12]	Deficiency of this protein contributes to inflammation and vascular damage in the kidney, a hallmark of glomerulonephritis [28].
EGFR	↓ [12]	EGFR is upregulated and activated in the synovial tissue of patients with autoimmune arthritis [29]. EGFR inhibition has been proposed as a therapeutic strategy for arthritis [29].
F2	↓ [12]	Thrombin activity is elevated in the spinal cord of animals with experimental autoimmune encephalomyelitis [30].
MASP1	↓ [12]	MASP1 contributes to the development and pathogenesis of lupus-like glomerulonephritis and rheumatoid arthritis [31,32].
MMP2	↑ [12]	MMP2 is upregulated in the plasma of patients with rheumatoid arthritis and systemic lupus erythematosus compared with controls [33].
SERPING1	↓ [11]	Plasma protease C1 inhibitor is involved in the complement cascade, and mutations in its encoding gene have been identified in patients with hereditary angioedema [34].
VWF	↓ [11]	Elevated VWF levels reflect systemic inflammation in patients with systemic lupus erythematosus [35].

*Note.* Full protein names and their relevant HGNC symbols (used here as protein identifiers): ADAMTS13, A disintegrin and metalloproteinase with thrombospondin type 1 motif, member 13; ADIPOQ, adiponectin; ALB, albumin; AGT, angiotensinogen; C5, complement component 5; C7, complement component 7; C9, complement component 9; C1S, complement C1s subcomponent; CFD, complement factor D; COL18A1, collagen alpha-1(XVIII) chain; EGFR, epidermal growth factor receptor; F2, coagulation factor II, thrombin; MASP1, mannan-binding lectin serine protease 1; MMP2, matrix metallopeptidase 2; SERPING1, plasma protease C1 inhibitor; VWF, von Willebrand factor.

a Protein identifiers are presented using HGNC gene symbols, which are commonly used in proteomics and bioinformatics databases such as STRING or PHENOPEDIA. Although these are gene symbols, they are used here to refer to proteins and are therefore presented in non-italicized uppercase.

b Indicates proteins differently expressed in centenarians compared to controls in both proteomic studies [11,12].

Albumin (ALB), significantly elevated in the plasma of centenarians compared to controls (Table 1) [11], emerged as the most connected hub protein, showing the highest node degree (ranging from 1 to 12, Supplementary File 1). Although ALB was not directly annotated as AD-related in the databases, it is known for its antioxidant and anti-inflammatory properties [36], and evidence suggests an inverse association between blood levels of this protein and the severity of certain ADs (including systemic lupus erythematosus, myasthenia gravis or neuromyelitis optica spectrum disorder) [[19], [20], [21]]. These observations support an overall potential protective role of ALB in centenarians.

Eight of the identified autoimmunity-related proteins—ADIPOQ, C1S, C5, C7, C9, CFD, MASP1, and SERPING1—belonged to the complement system (highlighted as colored nodes in Fig. 1). The strong representation of complement proteins, which have been implicated in various ADs [37,38], suggests a key role of this system in modulating immune responses and promoting resistance to these conditions in centenarians. In addition, several autoimmunity-related proteins are linked to coagulation (e.g., AGT, F2) and to specific ADs, such as systemic lupus erythematosus (e.g., AGT, C1S, C7, MMP2, VWF) or autoimmune rheumatic diseases (e.g., ADIPOQ, AGT, MASP1, MMP2) (Table 1).

## 4 Discussion

Our findings underline, at least in part, a potential immunomodulatory and protective role of ALB against the development of ADs in centenarians. Elevated ALB levels have been associated with lower AD severity [[19], [20], [21]] and improved response to immunotherapy [39,40], underscoring the regulatory function of this protein in inflammation and immune homeostasis. The identification of ALB as a central hub protein emphasizes its potential as a biomarker and therapeutic target for enhancing resistance to autoimmunity in aging populations.

The substantial presence of complement proteins within our PPI network further illustrates their dual capability to both promote inflammatory responses and establish regulatory conditions. Precise modulation of complement pathways could thus represent a crucial factor in centenarians' immune robustness, minimizing AD risk despite the presence of autoantibodies. Future research should investigate these regulatory interactions in detail, potentially revealing novel therapeutic avenues.

The exploratory nature of our study and its reliance on previously collected data represent primary limitations, emphasizing the need for prospective validation in larger and more diverse cohorts. Nonetheless, our findings offer preliminary insights into potential proteomic signatures underlying resistance to ADs in extreme longevity.

## 5 Conclusion

This report identifies ALB and Complement proteins as potential protective factors against ADs in centenarians, providing preliminary insights into proteomic mechanisms underpinning healthy aging and resistance to autoimmunity.

## CRediT authorship contribution statement

**Pedro Carrera-Bastos:** Writing – review & editing, Writing – original draft, Validation, Conceptualization. **Abel Plaza-Florido:** Writing – review & editing, Writing – original draft, Visualization, Validation, Software, Methodology, Investigation, Formal analysis, Conceptualization. **Alejandro Santos-Lozano:** Writing – review & editing, Validation, Methodology, Investigation, Formal analysis, Data curation. **Vânia Borba:** Writing – review & editing, Validation. **Gabriel Rodríguez-Romo:** Writing – review & editing. **Celia García-Chico:** Writing – review & editing, Visualization. **Simone Lista:** Writing – review & editing. **Gonzalo Saco-Ledo:** Writing – review & editing. **Enzo Emanuele:** Writing – review & editing, Validation, Supervision, Funding acquisition, Conceptualization. **Yehuda Shoenfeld:** Writing – review & editing, Validation, Supervision. **Alejandro Lucia:** Writing – review & editing, Validation, Supervision, Resources, Project administration, Funding acquisition, Conceptualization.

## Ethics statement

This study involved secondary analyses of publicly available proteomic data. No new human participants were enrolled, and ethical approval was not required.

## Data statement

The data underlying this article will be made available upon reasonable request to the corresponding author, subject to appropriate institutional review board approval.

## Declaration of competing interest

The authors declare no known competing financial interests or personal relationships that could have appeared to influence the work reported in this paper. Alejandro Lucia and Celia Garcia-Chico received research support from the World Cancer Research Fund and the European University Miguel de Cervantes, respectively. This support did not influence the design, analysis, or interpretation of the current study.

## Data Availability

Data will be made available on request.

## References

[1] Pisetsky D.S. (2023). Pathogenesis of autoimmune disease. Nat. Rev. Nephrol..

[2] Franceschi C., Garagnani P., Vitale G., Capri M., Salvioli S. (2017). Inflammaging and “Garb-aging,”. Trends Endocrinol. Metabol..

[3] Zheng Y., Liu Q., Goronzy J.J., Weyand C.M. (2023). Immune aging - a mechanism in autoimmune disease. Semin. Immunol..

[4] Valentino T.R., Chen N., Makhijani P., Khan S., Winer S., Revelo X.S., Winer D.A. (2024). The role of autoantibodies in bridging obesity, aging, and immunosenescence. Immun. Ageing.

[5] Yu F., Chen H., Li Q., Tao M., Jin Z., Geng L., Sun L. (2023). Secular trend of mortality and incidence of rheumatoid arthritis in global ,1990-2019: an age period cohort analysis and joinpoint analysis. BMC Pulm. Med..

[6] Thiruvengadam S., Luthra P. (2021). Thyroid disorders in elderly: a comprehensive review. Dis Mon.

[7] Goyne C.E., Fair A.E., Sumowski P.E., Graves J.S. (2024). The impact of aging on multiple sclerosis. Curr. Neurol. Neurosci. Rep..

[8] Anaya J.-M., Lozada-Martinez I.D., Torres I., Shoenfeld Y. (2024). Autoimmunity in centenarians. A paradox. Journal of Translational Autoimmunity.

[9] Zhou L., Ge M., Zhang Y., Wu X., Leng M., Gan C., Mou Y., Zhou J., Valencia C.A., Hao Q., Zhu B., Dong B., Dong B. (2022). Centenarians alleviate inflammaging by changing the ratio and secretory phenotypes of T helper 17 and regulatory T cells. Front. Pharmacol..

[10] Greenblatt J.F., Alberts B.M., Krogan N.J. (2024). Discovery and significance of protein-protein interactions in health and disease. Cell.

[11] Santos-Lozano A., Valenzuela P.L., Llavero F., Lista S., Carrera-Bastos P., Hampel H., Pareja-Galeano H., Gálvez B.G., López J.A., Vázquez J., Emanuele E., Zugaza J.L., Lucia A. (2020). Successful aging: insights from proteome analyses of healthy centenarians. Aging (Albany NY).

[12] Reed E.R., Chandler K.B., Lopez P., Costello C.E., Andersen S.L., Perls T.T., Li M., Bae H., Soerensen M., Monti S., Sebastiani P. (2025). Cross-platform proteomics signatures of extreme old age. Geroscience.

[13] Baranzini S.E. (2014). Symposium 2-1 the autoimmunome: similarities and differences among genetic susceptibility to common immune-related diseases. Nihon Rinsho Meneki Gakkai Kaishi.

[14] Szklarczyk D., Kirsch R., Koutrouli M., Nastou K., Mehryary F., Hachilif R., Gable A.L., Fang T., Doncheva N.T., Pyysalo S., Bork P., Jensen L.J., von Mering C. (2023). The STRING database in 2023: protein-protein association networks and functional enrichment analyses for any sequenced genome of interest. Nucleic Acids Res..

[15] Yu W., Clyne M., Khoury M.J., Gwinn M. (2010). Phenopedia and Genopedia: disease-centered and gene-centered views of the evolving knowledge of human genetic associations. Bioinformatics.

[16] Lu K., Liu L., Xu X., Zhao F., Deng J., Tang X., Wang X., Zhao B.-Q., Zhang X., Zhao Y. (2020). ADAMTS13 ameliorates inflammatory responses in experimental autoimmune encephalomyelitis. J. Neuroinflammation.

[17] Thomas M.R., de Groot R., Scully M.A., Crawley J.T.B. (2015). Pathogenicity of anti-ADAMTS13 autoantibodies in acquired thrombotic thrombocytopenic purpura. EBioMedicine.

[18] Brezovec N., Perdan-Pirkmajer K., Čučnik S., Sodin-Šemrl S., Varga J., Lakota K. (2021). Adiponectin deregulation in systemic autoimmune rheumatic diseases. Int. J. Mol. Sci..

[19] Yip J., Aghdassi E., Su J., Lou W., Reich H., Bargman J., Scholey J., Gladman D.D., Urowitz M.B., Fortin P.R. (2010). Serum albumin as a marker for disease activity in patients with systemic lupus erythematosus. J. Rheumatol..

[20] Weng Y.-Y., Yang D.-H., Qian M.-Z., Wei M.-M., Yin F., Li J., Li X., Chen Y., Ding Z.-N., He Y.-B., Zhang X. (2016). Low serum albumin concentrations are associated with disease severity in patients with myasthenia gravis. Medicine (Baltim.).

[21] Yao X.-Y., Wu Y.-F., Gao M.-C., Hong R.-H., Ding J., Hao Y., Zhang Y., Guan Y.-T. (2020). Serum albumin level is associated with the severity of neurological dysfunction of NMOSD patients. Mult Scler Relat Disord.

[22] Chang Y., Wei W. (2015). Angiotensin II in inflammation, immunity and rheumatoid arthritis. Clin. Exp. Immunol..

[23] Chu C.-Q. (2023). Complement-targeted therapy for autoimmune diseases. Mediev. Rev..

[24] Balduit A., Bianco A.M., Mangogna A., Zicari A.M., Leonardi L., Cinicola B.L., Capponi M., Tommasini A., Agostinis C., d'Adamo A.P., Bulla R. (2023). Genetic bases of C7 deficiency: systematic review and report of a novel deletion determining functional hemizygosity. Front. Immunol..

[25] Horiuchi T., Tsukamoto H., Sawabe T., Harashima S., Morita C., Kashiwagi Y., Himeji D., Masumoto K., Otsuka T., Kusaba T., Nagasawa K. (2000). Behçet’s disease associated with complement component 9 (C9) deficiency. Mod. Rheumatol..

[26] Ugarte-Berzal E., Martens E., Boon L., Vandooren J., Blockmans D., Proost P., Opdenakker G. (2019). EDTA/gelatin zymography method to identify C1s versus activated MMP-9 in plasma and immune complexes of patients with systemic lupus erythematosus. J. Cell Mol. Med..

[27] Barratt J., Weitz I. (2021). Complement factor D as a strategic target for regulating the alternative complement pathway. Front. Immunol..

[28] Hamano Y., Okude T., Shirai R., Sato I., Kimura R., Ogawa M., Ueda Y., Yokosuka O., Kalluri R., Ueda S. (2010). Lack of collagen XVIII/endostatin exacerbates immune-mediated glomerulonephritis. J. Am. Soc. Nephrol..

[29] Swanson C.D., Akama-Garren E.H., Stein E.A., Petralia J.D., Ruiz P.J., Edalati A., Lindstrom T.M., Robinson W.H. (2012). Inhibition of epidermal growth factor receptor tyrosine kinase ameliorates collagen-induced arthritis. J. Immunol..

[30] Göbel K., Eichler S., Wiendl H., Chavakis T., Kleinschnitz C., Meuth S.G. (2018). The coagulation factors fibrinogen, thrombin, and factor XII in inflammatory disorders-A systematic review. Front. Immunol..

[31] Machida T., Sakamoto N., Ishida Y., Takahashi M., Fujita T., Sekine H. (2018). Essential roles for mannose-binding lectin-associated serine protease-1/3 in the development of lupus-like glomerulonephritis in MRL/lpr mice. Front. Immunol..

[32] Banda N.K., Acharya S., Scheinman R.I., Mehta G., Coulombe M., Takahashi M., Sekine H., Thiel S., Fujita T., Holers V.M. (2016). Mannan-binding lectin-associated serine protease 1/3 cleavage of pro-factor D into factor D in vivo and attenuation of collagen antibody-induced arthritis through their targeted inhibition by RNA interference-mediated gene silencing. J. Immunol..

[33] Chang Y.-H., Lin I.-L., Tsay G.J., Yang S.-C., Yang T.-P., Ho K.-T., Hsu T.-C., Shiau M.-Y. (2008). Elevated circulatory MMP-2 and MMP-9 levels and activities in patients with rheumatoid arthritis and systemic lupus erythematosus. Clin. Biochem..

[34] Firinu D., Colomba P., Manconi P.E., Barca M.P., Fenu L., Piseddu G., Zizzo C., Del Giacco S.R., Duro G. (2013). Identification of a novel and recurrent mutation in the SERPING1 gene in patients with hereditary angioedema. Clin Immunol.

[35] Nossent J.C., Raymond W.D., Eilertsen G.Ø. (2016). Increased von Willebrand factor levels in patients with systemic lupus erythematosus reflect inflammation rather than increased propensity for platelet activation. Lupus Sci Med.

[36] Ward E.S., Gelinas D., Dreesen E., Van Santbergen J., Andersen J.T., Silvestri N.J., Kiss J.E., Sleep D., Rader D.J., Kastelein J.J.P., Louagie E., Vidarsson G., Spriet I. (2022). Clinical significance of serum albumin and implications of FcRn inhibitor treatment in IgG-mediated autoimmune disorders. Front. Immunol..

[37] Defendi F., Thielens N.M., Clavarino G., Cesbron J.-Y., Dumestre-Pérard C. (2020). The immunopathology of complement proteins and innate immunity in autoimmune disease. Clin. Rev. Allergy Immunol..

[38] Coss S.L., Zhou D., Chua G.T., Aziz R.A., Hoffman R.P., Wu Y.L., Ardoin S.P., Atkinson J.P., Yu C.-Y. (2023). The complement system and human autoimmune diseases. J. Autoimmun..

[39] Jang Y., Lee S.-T., Kim T.-J., Jun J.-S., Moon J., Jung K.-H., Park K.-I., Chu K., Lee S.K. (2018). High albumin level is a predictor of favorable response to immunotherapy in autoimmune encephalitis. Sci. Rep..

[40] Zheng M. (2022). Serum albumin: a pharmacokinetic marker for optimizing treatment outcome of immune checkpoint blockade. J. Immunother. Cancer.

